# Cost-effectiveness analysis of third-generation heat and moisture exchangers in patients who underwent laryngectomy in Japan

**DOI:** 10.1186/s12962-025-00662-4

**Published:** 2025-10-14

**Authors:** Nobuhiko Oridate, Thea Smedby, Chiara Ruzza, Michaela Roth, Mansi Mehta, Yoko Akachi, Rasmus Skovgaard, Takatoshi Itagaki

**Affiliations:** 1 Department of Otolaryngology-Head & Neck Surgery, Graduate School of Medicine, Yokohama City University, Yokohama, Japan; 2 Payer & Evidence, Coloplast A/S, Humlebaek, Denmark; 3 Atos Medical AB, Malmö, Sweden; 4 IQVIA, Bangalore, India; 5 IQVIA Solutions G.K, Tokyo, Japan; 6 Voice & Respiratory Care BU, Coloplast K.K, Tokyo, Japan

**Keywords:** Heat and moisture exchangers, Laryngectomy, Cost-effectiveness analysis, Markov model, Health outcomes, Quality-adjusted life year

## Abstract

**Background:**

National health insurance coverage for third-generation heat and moisture exchanger (HME) was approved in Japan for patients after total laryngectomy (TL). Our study evaluated the cost-effectiveness of third-generation HMEs (Provox^®^ Life™ such as Provox^®^ Life Night, Go, Home, Energy, and Protect) versus second-generation HMEs and no HME for patients who underwent TL in Japan.

**Methods:**

A Markov model with five health states was developed using an existing model from a Japanese public healthcare payer perspective, with a time horizon of 10 years and a cycle length of one year. Primary outcome was incremental cost-effectiveness ratio (ICERs), while secondary outcomes were pulmonary infection, mucus plug event and skin irritation. One-way sensitivity analyses (OWSA) and Probabilistic Sensitivity Analysis (PSA) were also conducted to determine the robustness of model conclusions.

**Results:**

Third-generation HMEs resulted in an ICER of JPY 2,350,010 per QALY gained, at a willingness-to-pay threshold of JPY 5,000,000 per QALY gained, as compared to second-generation HMEs, indicating cost-effectiveness. Similarly, with an ICER of JPY 4,708,917 per QALY gained, third-generation HMEs were also more cost-effective than no HME. Third-generation HMEs showed a decrease in pulmonary infections (average amounts of pulmonary infections per patient over 10 years: 0.26 vs. 0.39 and 0.55), mucus plug event (0.29 vs. 0.47 and 2.14) and skin irritation (1.80 vs. 2.78 and 0.00) compared to second-generation HMEs and no HME. According to OWSA, the transition probabilities between health states were the main drivers for the cost difference between third-generation and second-generation HMEs. PSA confirmed the robustness of the findings.

**Conclusion:**

This is the first study to evaluate cost-effectiveness of HMEs in Japan, suggesting that third-generation HMEs (Provox^®^ Life™) are more cost-effective compared to second-generation HMEs and no HME for patients who underwent TL in Japan.

**Supplementary Information:**

The online version contains supplementary material available at 10.1186/s12962-025-00662-4.

## Background

Total laryngectomy (TL) is the last surgical treatment option for patients with laryngeal cancer involving removal of the entire larynx and redirection of the trachea to a neck stoma [1, 2]. According to the National Malignancy Registry Report 2020 published by the Japanese Head and Neck Cancer Association, 14,837 adults were newly diagnosed with head and neck cancer in 2020 in Japan, of which, 1138 underwent laryngectomy [3]. In general, among patients who underwent TL, major pulmonary issues, such as coughing, excessive sputum production, and frequent forced expectorations were reported [4, 5]. These pulmonary changes have been associated with anxiety, depression, and disruptions to aspects of daily functioning including sleep and fatigue [4, 5].

The heat and moisture exchangers (HMEs) are the medical devices that compensate some of the functions that are lost after TL, and are attached over the stoma via an attachment (tube or adhesive) [6]. The use of HMEs by patients who underwent TL helps alleviate post-laryngectomy pulmonary problems [7, 8] including improved mucociliary clearance [9], reduced pulmonary infections [10], mucus plug incidence [11, 12], involuntary coughing, and forced expectorations [7, 8, 13, 14]. HMEs have also shown to significantly improve psychological wellbeing [8, 14, 15].

Since the 1990´s, a series of first- (Normal and HiFlow) and second-generation Provox^®^ XtraHMEs (XtraMoist™ and XtraFlow™) have been developed, which have shown continuous improvement in humidification, breathability and usability [6, 16–20]. Furthermore, the third-generation HMEs (Provox^®^ Life™ such as Provox^®^ Life Night, Go, Home, Energy, and Protect) have shown to provide enhanced humidification and breathability for patients following TL [14, 21, 22]. These devices have interchangeable options and improved adhesives, allowing for adaptability to patients’ daily routine and clinical needs [14, 21, 22].

Previously published studies have reported improvements in pulmonary symptoms with the use of third-generation HMEs, including significant reductions in daily forced expectoration, dry cough, shortness of breath, skin irritation, mucus plug events, and mucus production, as compared to second-generation HMEs [14, 21, 22]. Additionally, a significant improvement in the frequency and severity of cough and sputum, and their impact on daily activity (as assessed by Cough and Sputum Assessment Questionnaire [CASA-Q]), as well as a significant improvement in anxiety/depression (as assessed by EuroQol-5-dimensions [EQ-5D]) was observed with the use of third-generation HMEs [14, 22]. Contrarily, there were no significant changes in the overall quality of life (QoL) with the use of third-generation HMEs in existing HME users post-laryngectomy, as determined by EQ-5D-5L index [14, 22].

Till date, there are two cost-effectiveness and two cost-analysis studies of second-generations HMEs [12, 16, 23, 24]. We have not been able to identify any published health economic analysis of third-generation HMEs. National health insurance coverage for third-generation HMEs has been approved in Japan, making it available for users in the country as of November 2024. Its demonstrated cost-effectiveness is anticipated to support broader adoption by both patients and healthcare professionals. Our study aimed to assess the cost-effectivenss of third-generation HMEs (Provox Life), compared to second-generation and no HMEs, in patients with TL from a Japanese public healthcare payer perspective.

The objectives of this study were: (1) to investigate whether the third-generation HMEs and adhesives are cost-effective compared to second-generation HMEs and adhesives (current standard of care) and no HME for patients who underwent TL in Japan; and (2) to investigate differences in health outcomes (quality-adjusted life year [QALY], pulmonary infection, mucus plug events, and skin irritation) in patients who underwent TL using third-generation HMEs, second-generation HMEs, and no HME.

## Methods

### Model description

**Fig. 1:**
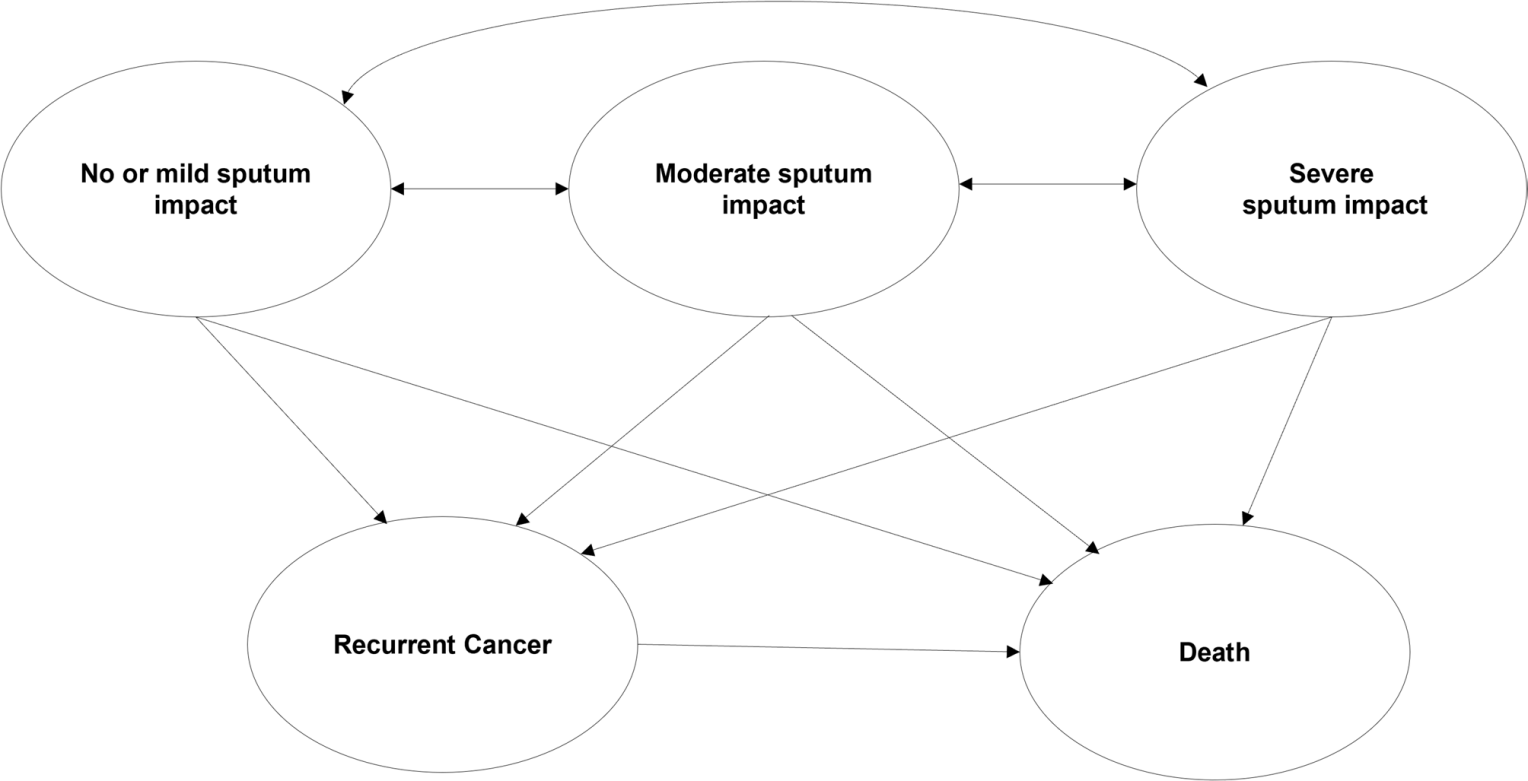
Model structure

A Markov model with five health states (Fig. 1) was developed using an existing model from a Japanese public healthcare payer perspective, with a time horizon of 10 years and a cycle length of one year. The 10 year time horizon was chosen based on the average age of TL patients and their average overall survival [25–28].The model simulated the course of events in a hypothetical cohort of 1000 patients with an average age of 67 years [14] with laryngeal cancer who underwent TL. All costs (from 2023) were reported in Japanese Yen (JPY) and an annual discount rate of 2.0% was applied to both cost and health outcomes. The model used the CASA-Q to identify health states that distinguish in health-related quality of life (HRQoL), or utility to better capture improvements in HRQoL in patients who underwent TL than cancer-based health states [29]. Patient-level data from Longobardi et al. study [14] was used to determine whether the CASA-Q can inform health states that distinguish in HRQoL. As per the Longobardi et al. study [14], the EQ-5D and CASA-Q were administered in 40 patients with three different measurement moments (i.e., 120 measurement moments). The cut-off scores CASA-Q were captured from the literature as follows: no or mild sputum impact (SPUI): ≥94, moderate SPUI: 64 ≤ 94, and severe SPUI: <64 [30].

### Treatments

Intervention third-generation HMEs were compared with second-generation HMEs and no HME.

### Model parameters and data source

Model inputs were updated through (a) review of an existing cost-effectiveness model, (b) validation by Japanese key opinion leaders (KOLs) who are experts on laryngectomy and health insurance, and (c) a target literature review for Japan-specific parameters.

### Expert consultation process

To supplement gaps in published Japanese cost data, structured interviews were conducted with two KOLs in Japan. Both experts possess clinical expertise in laryngectomy and a deep understanding of the national healthcare system and reimbursement policies. During the interviews, international data sources [23] were presented—such as estimates from the Portuguese model for “No HME” and other event costs—along with their Japanese Yen conversions. The KOLs reviewed these estimates for contextual accuracy.

A detailed resource table was used to obtain unit prices and monthly usage for relevant materials (e.g. foam pads, bibs, suction systems, nebulizers). Pre-identified cost inputs from local sources were validated during the consultation. Manufacturer purchase data for the Provox Protector bib were also analyzed to triangulate annual cost estimates.

The consultation extended beyond the “No HME” health state to include recurrent throat cancer, infection-related events, and other adverse events. For each, the KOLs were presented with relevant international estimates, local data where available, and asked to provide expert input on the most appropriate estimation approach for the Japanese setting.

A second KOL independently reviewed and validated the full set of inputs and assumptions. A targeted literature review was also conducted; however, no relevant Japanese publications were identified for the cost items considered.

### Targeted literature search

A search strategy in English language (Supplementary Table 1) was designed to search evidence for HMEs in TL. The parameters of interest were cost, QALYs and utility/disutility (Supplementary Table 2). Japan was the geography of interest. The search strategy retrieved 279 citations for title/abstract screenings. Of these, no citations were included in the screenings for next level. Hand searching was performed using Google search engine to supplement the search. However, no evidence was generated.

### Characteristics of study population

Starting point cohort (Table 1) was derived from a previously published study [14]. Patients who underwent TL and routinely used second-generation HMEs and adhesives were selected [14].

**Table 1:** Starting point cohort

Inputs	Proportion	Source
**Starting point cohort**		
No to mild SPUI	0.075	(14)
Moderate SPUI	0.525	
Severe SPUI	0.400	

Abbreviations: SPUI, sputum impact

### Clinical parameters and transition probabilities

Transition probabilities were estimated using an empirical, non-parametric approach based on anonymized patient-level data from a randomized cross-over study by Longobardi et al. (2022) [14]. Specifically, the probability of transitioning from one health state to another was calculated as the proportion of patients who moved from state A to state B, relative to the total number of patients initially in state A. This direct estimation method allowed us to reflect observed transitions without relying on parametric assumptions such as exponential functions.

The clinical parameters used in the model were primarily derived from this cross-over trial with random allocation to sequence, which evaluated pulmonary outcomes associated with different HME devices in post-laryngectomy patients. Additional parameters, where required, were supplemented from published literature and expert clinical input. Background mortality was used throughout the model, based on the Japanese life table for 2022 [31] and is described in Supplementary Table 3. Transition probabilities for risk of recurrent throat cancer and risk of death due to other causes were variable and were based on literature [32]. Transition probabilities for risk of death with recurrent throat cancer were inferred from a previous clinical trial [33]. These are described in Supplementary Table 4.

### Economic parameters

Economic parameters are described in Table 2. Health state costs base-case for second- and third-generation HMEs were calculated based on the HMEs costs and the assumptions of their usage based on each health state. The no HME cost was based on an estimate provided by KOLs. Costs for the use of HMEs were provided by the manufacturer as the set price for each part of HMEs. Event costs for skin irritation were provided by the KOLs.

**Table 2:** Economic input parameters

Health state costs base-case	Cost	Source
**Third-generation HMEs**		
No or mild SPUI	¥681,191	(14)
Moderate SPUI	¥397,860	(14)
Severe SPUI	¥449,905	(14)
**Second-generation HMEs**		
No or mild SPUI	¥332,632	(14)
Moderate SPUI	¥324,196	(14)
Severe SPUI	¥359,406	(14)
**No HME**		
No or mild SPUI	¥30,000	KOLs
Moderate SPUI	¥30,000	KOLs
Severe SPUI	¥30,000	KOLs
Recurrent throat cancer	¥7,435,931	(33)
Death (end-of-life care)	¥1,297,316	(35)
**Event costs**		
Skin irritation	¥150	KOLs
Mucus plug event	¥27,500	(35)
Pulmonary infection	¥451,754	(34)

Abbreviations: HME, heat and moisture exchanger; KOL, key opinion leader; SPUI, sputum impact; ¥, Japanese Yen

Event costs for mucus plug event and pulmonary infection were inferred from the published literature [34, 35] (Table 2). Cost of infection included infection by non-bacteremic pneumococcal pneumonia, and it was inferred from previously published study [34] (Supplementary Table 5). Invasive pneumococcal disease was excluded based on expert advice, as it is a rare but severe complication. To adopt a conservative approach and avoid overestimating cost offsets, only non-bacteremic pneumococcal pneumonia was included. This includes the hospitalization and outpatient visit. The official medical fee revision rates were used, which is a common practice in Japan (instead of applying Consumer Price Index) (Supplementary Table 6).

### Utilities

HRQoL was modeled by assigning utilities to various health states, expressed in QALYs. QALYs were calculated by multiplying the life years gained times the utility scores. Health state utilities (base-case) for no to mild SPUI, moderate SPUI, severe SPUI, recurrent throat cancer and death were derived from an Italian study [14], since Japanese specific parameters do not exist (Table 3). Disutilities are utility decrements that reflect the adverse effect of a certain condition on a patient’s QoL by subtracting the disutility from the patient’s utility value (Table 3). In this analysis, disutilities were applied for skin irritation [36], pulmonary infection [34], and mucus plug event [36], specific to the Japanese population (Table 3).

**Table 3:** Model input parameters: utilities and disutilities

Health state utilities base-case	Utility	Source
No to mild SPUI	0.9183	(14)
Moderate SPUI	0.8009	
Severe SPUI	0.6228	
Recurrent throat cancer	0.6300	
Death	0.0000	
**Event disutilites**		
Skin irritation	0.0430	(36)
Pulmonary infection	0.0434	(34)
Mucus plug event	0.0660	(36)
**Health state utilities (including disutilities due to events)**		
**Third-generation HMEs**		
No to mild SPUI	0.8931	
Moderate SPUI	0.7757	
Severe SPUI	0.5976	
**Second-generation HMEs**		
No to mild SPUI	0.8791	
Moderate SPUI	0.7617	
Severe SPUI	0.5836	
**No HME**		
No to mild SPUI	0.8797	
Moderate SPUI	0.7623	
Severe SPUI	0.5842	

Abbreviations: HME, heat and moisture exchanger; SPUI, sputum impact; ¥, Japanese Yen

### Analysis of cost-effectiveness

The base-case analysis was conducted for the intervention (third-generation HMEs) and the two comparators (second-generation HME and no HME) and modeled primary outcomes were reported as incremental costs, incremental QALYs and incremental cost-effectiveness ratios (ICERs). The ICER was compared with the Japanese willingness-to-pay (WTP) threshold of JPY 5,000,000 per QALY gained to reflect on the primary outcomes. It is important to note that no official threshold of cost-effectiveness of medical interventions exist in Japan, however, JPY 5,000,000 per QALY gained is the most applied value [37]. This same threshold has been applied to a cost-effectiveness study of a medical device [38]. The secondary outcomes were reported as pulmonary infection, mucus plug event, and skin irritation.

### Scenario analysis

For the base-case analysis, conservative value of no HME health costs was used (JPY 30,000), as provided by the KOLs. Therefore, a scenario analysis was performed, wherein the cost for no HME was kept as JPY 170,000, as described in the Beck et al. 2020 method [23].

### One-way sensitivity analysis

Sensitivity analyses were conducted to account for uncertainties around the model parameters and determine the robustness of model conclusions. One-way sensitivity analyses (OWSA) were conducted to assess the impact of individual parameter uncertainty on the ICER. The analysis included a range of clinical and cost parameters, such as the probability of pulmonary infection, recurrent throat cancer mortality, mucus plug events, skin irritation, health state costs, end-of-life care costs, and costs associated with adverse events. These inputs were varied within plausible ranges to identify the most influential parameters. In case no lower and upper bound were reported in original research, the base-case value was increased and decreased by 20% to determine upper and lower bound values [35].

### Probabilistic sensitivity analysis

A probabilistic sensitivity analysis (PSA) was conducted using a Monte Carlo simulation with 2,000 iterations to assess the impact of parameter uncertainty on model outcomes. Probability distributions were assigned to all uncertain parameters based on their statistical properties and standard practice in health economic modeling. A Dirichlet distribution was used to model the initial distribution of the cohort across mutually exclusive health states. Beta distributions were applied to parameters bounded between 0 and 1, including utilities, transition probabilities, and the risk of adverse events. Gamma distributions were used for cost parameters. Where variability in the input parameters was not available from the literature or data sources, a 20% variation from the mean input value was assumed as a conservative approach. These choices are consistent with recommended practices in health economic evaluation guidelines, including those from ISPOR [39]. Results of the PSA were presented in the cost-effectiveness plane and the cost-effectiveness acceptability curve (CEAC). Confidence intervals for secondary outcomes were built from the distribution of results generated through the PSA.

## Results

### Primary outcomes

#### Third-generation vs. second-generation HMEs

Based on the available evidence, the model-based base-case analysis showed that third-generation HMEs improved the effect (QALY: 4.71 vs. 4.33) and increased the total healthcare spending per patient (JPY 22,922,149 vs. JPY 22,038,701) over 10 years compared to second-generation HMEs for patients who underwent TL in Japan. This amounted to an incremental QALY of 0.38 and an incremental cost of JPY 883,447, resulting in an ICER of JPY 2,350,010 per QALY gained, which was less than the WTP threshold value of JPY 5,000,000 per QALY gained. This indicates that third-generation HMEs was more cost-effective than the second-generation HMEs (Table 4).

**Table 4:** Model primary outcomes of cost-effectiveness of third-generation HMEs vs. comparators

	Third-generation HMEs	Second-generation HMEs	No HME
QALYs	4.7087	4.3328	4.3079
Total costs	¥22,922,149	¥22,038,701	¥21,034,644
**Incremental results (Third-generation HMEs versus comparators)**			
Incremental QALYs	NA	0.3759	0.4008
Incremental costs	NA	¥883,447	¥1,887,504
ICER	NA	¥2,350,010	¥4,708,917

Abbreviations: HME, heat and moisture exchanger; ICER, incremental cost-effectiveness ratio; QALY, quality-adjusted life year; ¥, Japanese Yen

#### Third-generation HMEs vs. no HME

As compared to no HME, third-generation HME improved the QALY (4.71 vs. 4.31) and increased the total healthcare spending per patient (JPY 22,922,149 vs. JPY 21,034,644) over 10 years, resulting in an incremental QALY and incremental cost of 0.40 and, JPY 1,887,504 respectively (Table 4). The resulting ICER (JPY 4,708,917 per QALY gained) was less than the WTP threshold value (JPY 5,000,000 per QALY gained), indicating that third-generation HMEs were more cost-effective than the no HME (Table 4).

### Secondary outcomes

#### Third-generation HMEs vs. second-generation HMEs

The use of third-generation HMEs was associated with lower rates of adverse events compared to second-generation HMEs. Over a 10-year period, the average number of pulmonary infections per patient was 0.26 (95% CI: 0.14–0.43) with third-generation HMEs versus 0.39 (95% CI: 0.24–0.56) with second-generation HMEs. Similarly, the incidence of mucus plug events was reduced (0.29 [95% CI: 0.15–0.49] vs. 0.47 [95% CI: 0.29–0.67]), as was skin irritation (1.80 [95% CI: 1.10–2.59] vs. 2.78 [95% CI: 1.60–3.95]) (Table 5).

**Table 5:** Secondary outcomes

HME type	Pulmonary infection (95% CI)^*^	Mucus plug event (95% CI)^*^	Skin irritation (95% CI)^*^
Third-generation HMEs	0.26 (0.14–0.43)	0.29 (0.15–0.49)	1.80 (1.10–2.59)
Second-generation HMEs	0.39 (0.24–0.56)	0.47 (0.29–0.67)	2.78 (1.60–3.95)
No HME	0.55 (0.35–0.81)	2.14 (1.32–2.97)	0.00 (0.00–0.00)

^*^Secondary outcomes over a 10-year time horizon, reported as mean values with 95% CI derived from PSA. CI represent the 2.5th and 97.5th percentiles of the simulated distributions

Abbreviations: CI, confidence interval; HME, heat and moisture exchanger; PSA, probabilistic sensitivity analysis

#### Third-generation HMEs vs. no HME

Compared to no HME, third-generation HMEs resulted in fewer pulmonary infections (0.26 [95% CI: 0.14–0.43] vs. 0.55 [95% CI: 0.35–0.81]) and mucus plug events (0.29 [95% CI: 0.15–0.49] vs. 2.14 [95% CI: 1.32–2.97]). However, skin irritation was more frequent with third-generation HMEs (1.80 [95% CI: 1.10–2.59]) compared to no HME (0.00 [95% CI: 0.00–0.00]) (Table 5). This result regarding skin irritation was expected as patients utilizing no HME do not use adhesives which may lead to skin irritation.

### One-way sensitivity analysis

#### Third-generation HMEs vs. second-generation HMEs

**Fig. 2:**
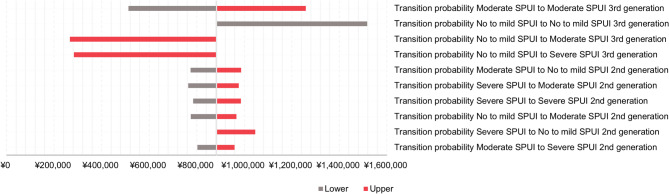
Tornado plots displaying most influential parameters from one-way sensitivity analyses for third-generation HMEs vs. second-generation HMEs. Abbreviations: HME, heat and moisture exchanger

Based on the sensitivity analyses, it was indicated that the transition probabilities between health states were the main drivers for the cost difference between third-generation and second-generation HMEs (Fig. 2**)**. This finding is consistent with the fact that the more severe SPUI was associated with higher cost and lower utility state.

#### Third-generation HMEs vs. no HME

**Fig. 3:**
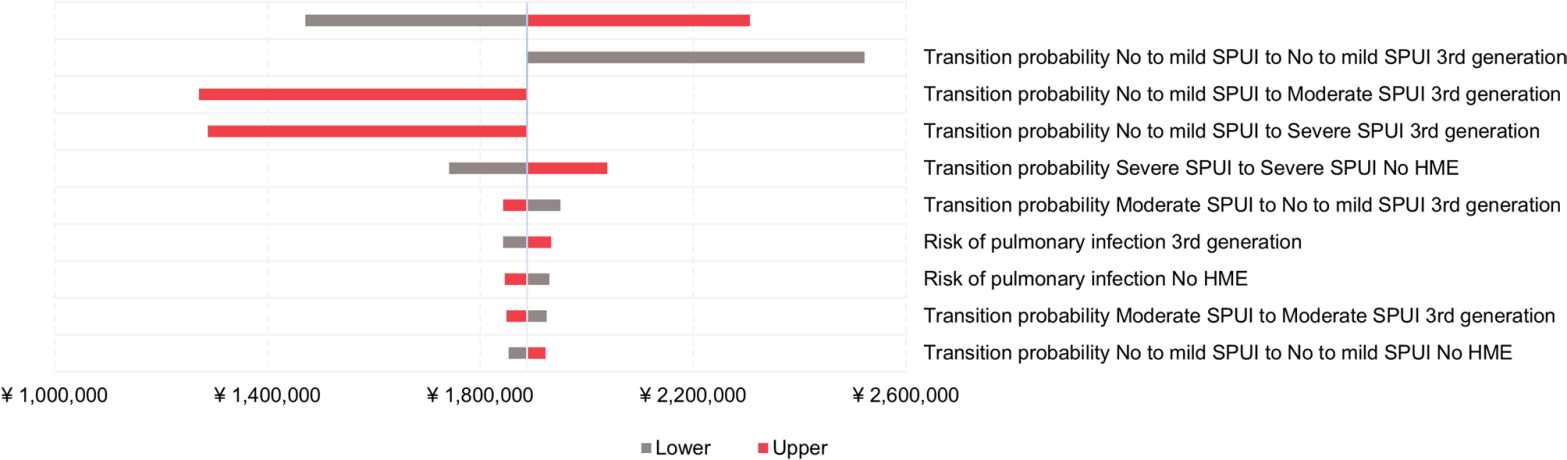
Tornado plots displaying most influential parameters from one-way sensitivity analyses for third-generation HMEs vs. no HME. Abbreviations: HME, heat and moisture exchanger

As shown in Fig. 3, transition probabilities between health states and the risk of pulmonary infections were the main drivers for the cost difference between third-generation HMEs and no HME.

### Scenario analysis for third-generation HMEs versus no HME: using JPY 170,000 as the cost for no HME

The scenario analysis showed that third-generation HMEs improved the effect (QALY: 4.71 vs. 4.31) and increased the total healthcare spending per patient (JPY 22,922,149 vs. JPY 21,541,961) over 10 years compared to no HME for patients who underwent TL in Japan. This amounted to an incremental QALY of 0.40 and an incremental cost of JPY 1,380,188, resulting in an ICER of JPY 3,443,271 per QALY gained, which was less than the WTP threshold value of JPY 5,000,000 per QALY gained. This indicates that third-generation HMEs were more cost-effective than the no HME (Table 6).

**Table 6:** Scenario analysis for third-generation HMEs vs. no HME: using JPY 170,000 as the cost for no HME

	Third-generation HMEs	No HME
QALYs	4.7087	4.3079
Total costs	¥22,922,149	¥21,541,961
Incremental QALYs	0.4008	
Incremental costs	¥1,380,188	
ICER	¥3,443,271	

Abbreviations: HME, heat and moisture exchanger; ICER, incremental cost-effectiveness ratio; QALY, quality-adjusted life year; ¥, Japanese Yen

**Fig. 4:**
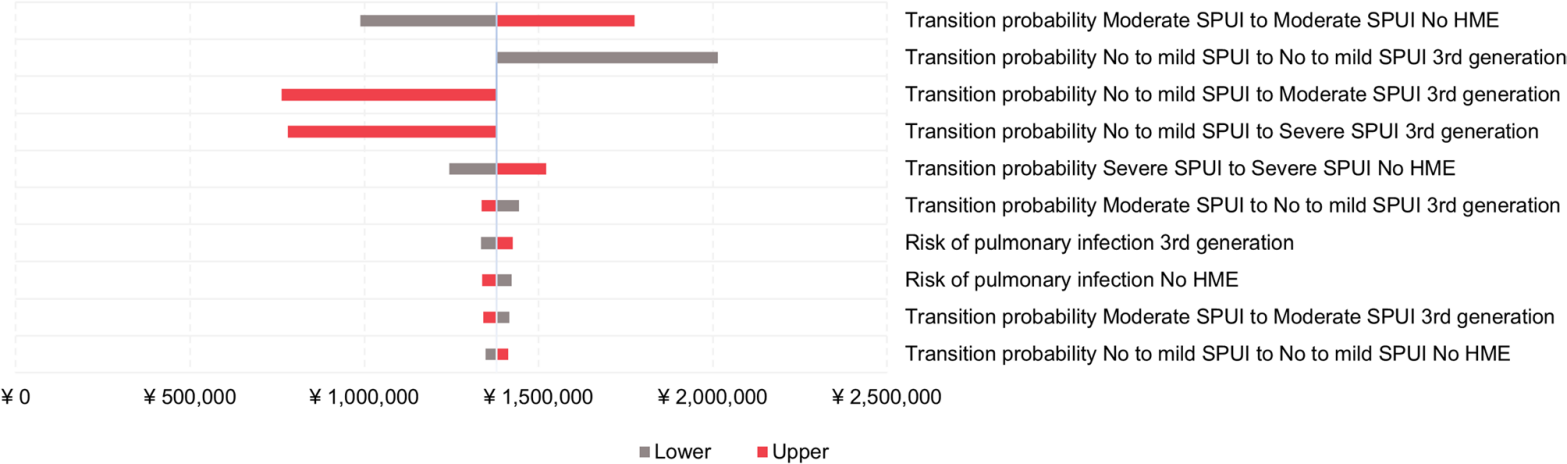
Tornado plots displaying most influential parameters from one-way sensitivity analyses for scenario analysis (third-generation HMEs vs. no HME). Abbreviations: HME, heat and moisture exchanger

Similar to base-case analysis, OWSA for scenario analysis showed that transition probabilities between health states and the risk of pulmonary infections were the main drivers for the cost difference between third-generation HMEs and no HME (Fig. 4).

### Probabilistic sensitivity analysis

**Fig. 5:**
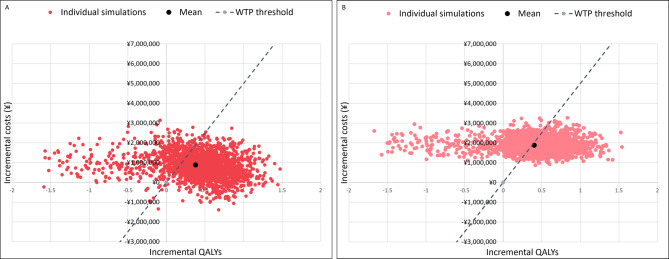
PSA cost-effectiveness scatter plots. **a**. Third-generation HMEs vs. second-generation HMEs. **b**. Third-generation HMEs vs. no HME. Abbreviations: HME, heat and moisture exchanger; PSA, probabilistic sensitivity analysis; QALY, quality-adjusted life year; WTP, willingness-to-pay

The PSA results are illustrated in three cost-effectiveness scatter plots **(**Fig. 5 and Supplementary Fig. 1). For third-generation HMEs vs. second-generation HMEs (Fig. 5a), most simulations cluster near the origin, with the mean also close to zero, indicating minimal differences in cost and QALYs. Some simulations fall below the WTP threshold, suggesting third-generation HMEs may be cost-effective in certain scenarios. The black dot represents the average incremental costs and QALYs of all simulations, which falls below the WTP threshold.

In the third-generation HMEs vs. No HME comparison (Fig. 5b), simulations were more widely spread, with many falling below the WTP threshold. The mean lay in the northeast quadrant, indicating that third-generation HMEs were generally more effective and more costly, but often remained within acceptable cost-effectiveness limits.

**Fig. 6:**
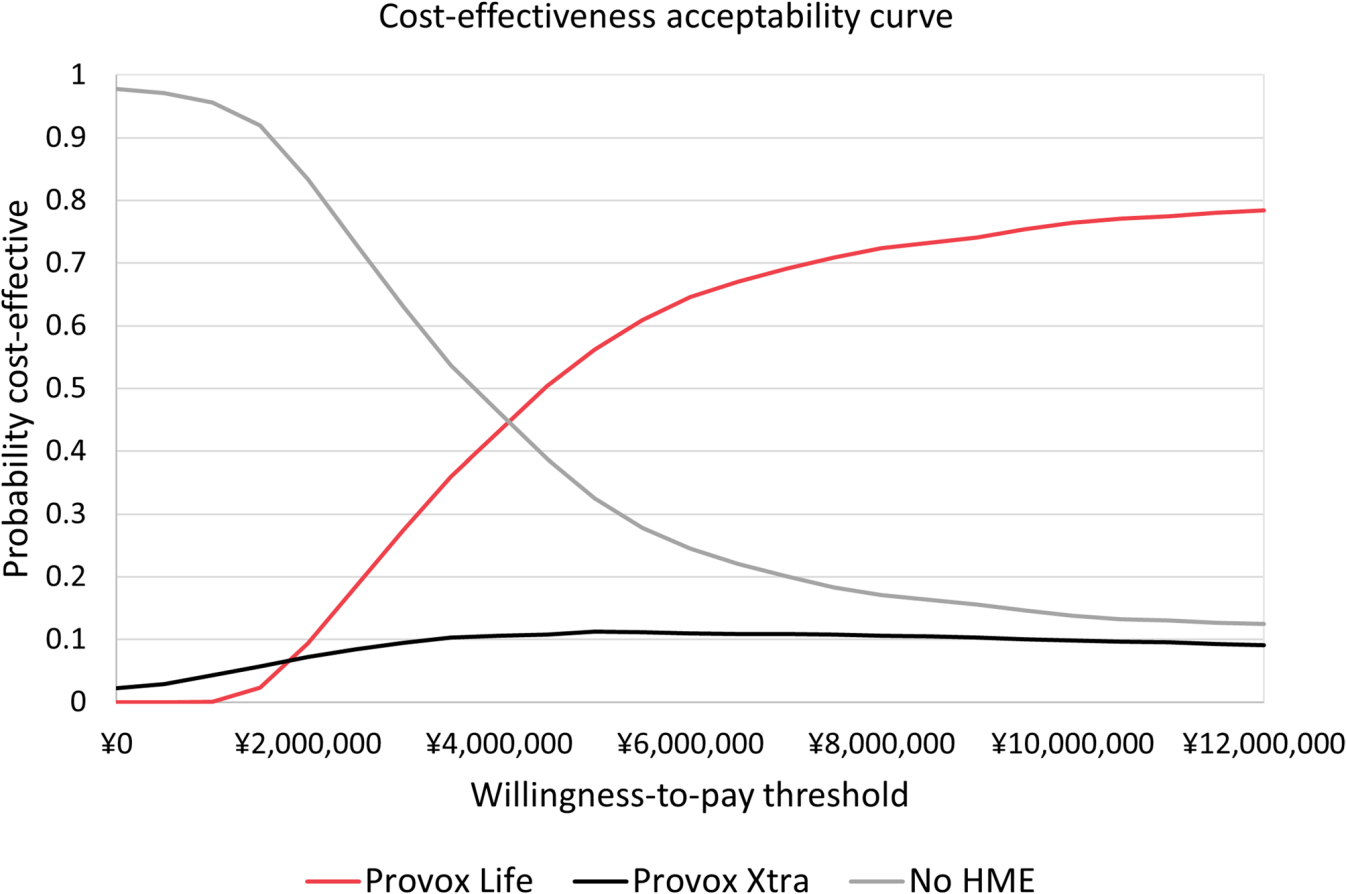
Cost-effectiveness acceptability curve. Abbreviations: HME, heat and moisture exchanger

The CEAC supported these findings. Third-generation HMEs showed increasing probability of being cost-effective with higher WTP, reaching ~ 75% at ¥12,000,000. Second-generation HMEs remained consistently low, while No HME started high but declined sharply, becoming negligible at higher thresholds (Fig. 6).

## Discussion

This is the first study to analyze the cost-effectiveness of third-generation HMEs compared with the second-generation HMEs and no HME in patients who underwent TL in Japan. Over a time horizon of 10 years, the results from our study demonstrated a substantial improvement in QALYs (0.38), along with an increase in the cost (JPY 883,447), with the use of third-generation compared to second-generation HMEs, resulting in an ICER of JPY 2,350,010 per QALY gained. Similarly, compared to no HME, third-generation HMEs showed an incremental QALY of 0.40 and an incremental cost of JPY 1,887,504, resulting in an ICER of JPY 4,708,917 per QALY gained. As a result, third-generation HMEs were found to be cost-effective compared to second-generation HMEs and no HME at a WTP threshold of JPY 5,000,000 per QALY gained.

At present, there are no published studies comparing cost-effectiveness of third-generation HMEs with second- or first-generation HMEs. Nevertheless, two studies conducted in United States and Poland demonstrated cost-effectiveness of second-generation HMEs compared to no HMEs (usual care/alternative stoma covers) [23, 24]. A study conducted in the United States from both societal and payer perspectives found that second-generation HMEs improved QALYs and were more effective and less costly compared to alternative stoma covers when evaluated over a lifetime horizon [23]. Similarly, a study conducted in Poland demonstrated that second-generation HMEs were more costly but cost-effective compared to usual care (including stoma covers, suction systems, and/or external humidifiers) in laryngectomized patients from a payer perspective over a 10-year time horizon [24]. In contrast, our analysis focused on third-generation HMEs, which were associated with higher costs but remained cost-effective relative to second-generation HMEs or no HME use. Several factors may explain these differences. First, the model structures differ; both the U.S. and Polish studies employed health states that were not derived from validated patient-reported outcomes, whereas our model used health states based on the CASA-Q instrument, which more accurately reflects the clinical and QoL outcomes relevant to total laryngectomized patients. Second, the scope of healthcare resource use included in the models varies. Our model adopted a conservative costing approach, incorporating only the cost of the devices per health state and the costs associated with three main adverse events. It did not include broader healthcare costs such as follow-up visits, medication use, or disease progression, which were considered in the U.S. and Polish models [23, 24]. This difference in cost accounting may explain why, in contrast to those studies where the no HME comparator was more costly than HME use, our model found that third-generation HMEs incurred higher costs.

In our model, broader healthcare costs such as follow-up visits, medication use, and disease progression were excluded due to the lack of reliable Japan-specific data and the structural specificities of the model. While this conservative costing approach avoids inflating cost-effectiveness estimates, it likely underestimates the full economic value of third-generation HMEs. We acknowledge this as a specificity of the model and recommend that future research incorporate these costs when appropriate data become available.

These differences underscore the importance of contextualizing cost-effectiveness results within the specific healthcare system, model structure, and input assumptions used. Cross-country comparisons should therefore be interpreted with caution, particularly given variations in WTP thresholds, utility inputs, and healthcare resource utilization. Our model also showed an improvement of number of pulmonary infections, mucus plug events and skin irritation with the use of third-generation HMEs versus second-generation HMEs. Third-generation HMEs resulted in a fewer pulmonary infections and mucus plug event, but led to increased skin irritation, compared to no HME. This data is in line with previously published studies conducted in Japan [40], Australia [22] and Germany [21], as described further. A prospective study conducted in Japan reported a significant improvement in pulmonary symptoms such as coughing and degree of dyspnea with the use of third-generation HMEs among existing HME users [40]. Another study conducted in Australia showed an improvement in pulmonary symptoms (SPUI, involuntary coughing, etc.) with the use of third-generation HMEs in existing HME users post-laryngectomy [22]. Furthermore, a study conducted in Germany showed a significant improvement in pulmonary symptoms such as daily forced expectoration, cough and sputum symptoms after the use of third-generation HMEs as compared to usual care, in patients who underwent TL [21].

The results of OWSA, as demonstrated by Tornado plots, illustrated the most influential input parameters. It was indicated that the transition probabilities between health states were the main drivers for the cost difference between third-generation and second-generation HMEs. Additionally, it was observed that the transition probabilities between health states were the main drivers for the cost difference between third-generation HMEs and no HME. Of note, the cost-effectiveness conclusion would change at a probability of 12.4% for patients remaining in the no or mild sputum health state when using third-generation HMEs, corresponding to an ICER equal to the WTP threshold of ¥5,000,000. However, from the available clinical data on Provox Life, such a variation is unlikely [14, 22].

This study has several strengths. This study is a novel contribution as it is the first study to evaluate the cost-effectiveness of third-generation HMEs compared to second-generation HMEs and no HMEs in Japan, filling an important gap in the literature. By adapting existing validated model to the Japanese healthcare context, the study provides practical insights to local healthcare.

It is also important to acknowledge few of the limitations of this study. Firstly, the results of this study must be interpreted in line with a few limitations related to the study design, particularly around the availability of inputs to populate the model; especially the information on pulmonary infections was based on assumptions and inputs from the KOLs. Secondly, there is a lack of published data specific to healthcare resource utilization and costs for patients with TL in Japan and globally, necessitating reliance on expert input and assumptions. Additionally, there is no direct comparative data evaluating outcomes between patients using no HME and those using third-generation HMEs, which limits the robustness of this comparison. The simplicity of the model, while advantageous for clarity and feasibility, may not fully reflect the complexity of real-world clinical pathways, patient variability, or detailed healthcare resource use. Additionally, uniform assumptions on costs and transitions may overlook subgroup differences, and simplified interactions between clinical outcomes could underestimate their overall impact on QoL and costs. Lastly, the utility values used in the model were sourced from an Italian study and may not accurately capture the preferences of the Japanese population. Future research should prioritize the collection of Japan-specific utility data—using instruments such as the EQ-5D or CASA-Q—to enhance the validity and cultural relevance of economic evaluations.

## Conclusion

Our results suggest that third-generation HMEs (Provox^®^ Life™) are more cost-effective as compared to second-generation HMEs and no HME, for patients who underwent TL in Japan from a public healthcare payer perspective. Third-generation HMEs also reduces the number of pulmonary infections, mucus plug events and skin irritation. In the future, collecting Japan-specific data on healthcare resource utilization, costs, and QoL measures for patients who underwent TL could refine the model and improve its accuracy. In addition, exploring the long-term real-world outcomes of third-generation HMEs through observational studies or registries could validate the modeled results. Conducting comparative analyses of advanced HMEs across different healthcare systems may also provide insights into best practices for implementation.

## Supplementary Information

Below is the link to the electronic supplementary material.

Supplementary Material 1

## Data Availability

The datasets generated during and/or analyzed during the current study are available from the corresponding author on reasonable request. All data supporting the findings of this study are available within the paper.

## Abbreviations

CASA-Q: Cough and Sputum Assessment Questionnaire
CEAC: Cost-Effectiveness Acceptability Curve
CI: Confidence Interval
CPI: Consumer Price Index
EQ-5D: EuroQol-5-dimensions
HME: Heat and moisture exchanger
HRQoL: Health-related quality of life
ICER: Incremental cost-effectiveness ratio
JPY: Japanese Yen
KOL: Key opinion leader
OWSA: One-way sensitivity analyses
PSA: Probabilistic sensitivity analysis
QALY: Quality-adjusted life year
QoL: Quality of life
SPUI: Sputum impact
TL: Total laryngectomy
WTP: Willingness-to-pay
