# Supplementary material for: Cost-effectiveness analysis of third-generation heat and moisture exchangers in patients who underwent laryngectomy in Japan
Source: Cost Eff Resour Alloc. 2025 Oct 14;23:54. doi: 10.1186/s12962-025-00662-4 (PMC12522815; doi:10.1186/s12962-025-00662-4)
Supplement: Supplementary file 1 — Supplementary Material 1 [file 12962_2025_662_MOESM1_ESM.docx]

# Cost-effectiveness Analysis of Third-generation Heat and Moisture Exchangers in Patients who underwent Laryngectomy in Japan

# Authors:

Nobuhiko Oridate ^1^, Thea Smedby^2^, Chiara Ruzza^3^, Michaela Roth^4^, Mansi Mehta^5^, Yoko Akachi^6^, Rasmus Skovgaard^7^, Takatoshi Itagaki^8^*

**Author Affiliations:**

Department of Otolaryngology-Head & Neck Surgery, Yokohama City University Graduate School of Medicine. noridate@yokohama-cu.ac.jp

Payer & Evidence, Coloplast A/S, Denmark. [dktsme@coloplast.com](mailto:dktsme@coloplast.com)

Payer & Evidence, Coloplast A/S, Denmark. dkchru@coloplast.com

Atos Medical AB, Sweden. Michaela.roth@atosmedical.com

IQVIA, Bangalore, India. mansi.mehta@iqvia.com

IQVIA Solutions G.K., Japan. yoko.akachi@iqvia.com

Payer & Evidence, Coloplast A/S, Denmark dkras@coloplast.com

Voice & Respiratory Care BU, Coloplast K.K., Japan. Kart.Itagaki@atosmedical.com

***Correspondence:**

Name: Takatoshi Itagaki

Affiliation: Voice & Respiratory Care BU Coloplast K.K., Japan

Tel: +81 8080209745

E-mail: [Kart.Itagaki@atosmedical.com](mailto:Kart.Itagaki@atosmedical.com)

# SUPPLEMENTARY INFORMATION

## Supplementary Table 1: Search strategy: Embase <1974 to 2024 October 29>/ Ovid MEDLINE(R) ALL <1946 to October 29, 2024>

| **S. No** | **Search string** | **# of hits** |
| --- | --- | --- |
| 1 | exp laryngectomy/ or laryngectom*.ti,ab. or Pharyngolaryngectomy.ti,ab. | 28085 |
| 2 | (((remov* or surg*) adj3 laryn*) or (heat adj3 moisture exchanger*) or HME* or sputum impact or spui).ti,ab. | 21432 |
| 3 | 1 or 2 | 46464 |
| 4 | exp Japan/ or Japan*.ti,ab. | 821280 |
| 5 | 3 and 4 | 379 |
| 6 | remove duplicates from 5 | 283 |
| 7 | limit 6 to (english or Japanese) | 279 |

## Supplementary Table 2: PICOS used for screening

| **Population** | Adult patients with laryngectomy |
| --- | --- |
| **Intervention/Comparator** | HME |
| **Outcomes** | Cost, QALY, Utility/Disutility |
| **Study design** | Economic evaluations, case control, cohort, cross-sectional studies |
| **Geography** | Japan |
| **Parameters of interest** | SPUI |

Abbreviations: HME, heat and moisture exchanger; SPUI, sputum impact; QALY, quality-adjusted life year

## Supplementary Table 3: Background mortality for Japanese population

| **Age** | **Index** | **Mortality** | **Source** |
| --- | --- | --- | --- |
| 67 | 0 | 0.008960 | [28] |
| 68 | 1 | 0.009910 |  |
| 69 | 2 | 0.011005 |  |
| 70 | 3 | 0.012215 |  |
| 71 | 4 | 0.013565 |  |
| 72 | 5 | 0.015085 |  |
| 73 | 6 | 0.016690 |  |
| 74 | 7 | 0.018390 |  |
| 75 | 8 | 0.020310 |  |
| 76 | 9 | 0.022500 |  |
| 77 | 10 | 0.025050 |  |
| 78 | 11 | 0.028050 |  |
| 79 | 12 | 0.031535 |  |
| 80 | 13 | 0.035345 |  |
| 81 | 14 | 0.039775 |  |
| 82 | 15 | 0.045045 |  |
| 83 | 16 | 0.051195 |  |
| 84 | 17 | 0.058265 |  |
| 85 | 18 | 0.066345 |  |
| 86 | 19 | 0.075525 |  |
| 87 | 20 | 0.085915 |  |
| 88 | 21 | 0.097740 |  |
| 89 | 22 | 0.110880 |  |
| 90 | 23 | 0.125215 |  |
| 91 | 24 | 0.141000 |  |
| 92 | 25 | 0.158490 |  |
| 93 | 26 | 0.178265 |  |
| 94 | 27 | 0.200625 |  |
| 95 | 28 | 0.225765 |  |
| 96 | 29 | 0.251520 |  |
| 97 | 30 | 0.278680 |  |
| 98 | 31 | 0.307230 |  |
| 99 | 32 | 0.337135 |  |
| 100 | 33 | 0.368340 |  |

## Supplementary Table 4: Transition probabilities for HMEs

| **Transition probabilities base-case** | **Value** | **Lower** | **Upper** | **Source** |
| --- | --- | --- | --- | --- |
| Risk of recurrent throat cancer | variable |  |  | [29] |
| Risk of death with recurrent throat cancer | 0.249 | 0.227 | 0.271 | [30] |
| Risk of death due to other causes | variable |  |  | [29] |
| **Third-generation HMEs** | | | | |
| No to mild SPUI to No to mild SPUI | 1.000 | 0.800 | 1.000 | [14] |
| No to mild SPUI to Moderate SPUI | 0.000 | 0.000 | 0.200 |  |
| No to mild SPUI to Severe SPUI | 0.000 | 0.000 | 0.200 |  |
| Moderate SPUI To No to Mild SPUI | 0.667 | 0.5336 | 0.8004 |  |
| Moderate SPUI to Moderate SPUI | 0.286 | 0.2288 | 0.3432 |  |
| Moderate SPUI to Severe SPUI | 0.048 | 0.0384 | 0.0576 |  |
| Severe SPUI to No to mild SPUI | 0.250 | 0.2 | 0.3 |  |
| Severe SPUI to Moderate SPUI | 0.688 | 0.5504 | 0.8256 |  |
| Severe SPUI to Severe SPUI | 0.063 | 0.0504 | 0.0756 |  |
| **Second-generation HMEs** | | | | |
| No to mild SPUI to No to mild SPUI | 0.333 | 0.2664 | 0.400 | [14] |
| No to mild SPUI to Moderate SPUI | 0.667 | 0.5336 | 0.800 |  |
| No to mild SPUI to Severe SPUI | 0 | 0 | 0.200 |  |
| Moderate SPUI to no to Mild SPUI | 0.190 | 0.152 | 0.228 |  |
| Moderate SPUI to Moderate SPUI | 0.667 | 0.5336 | 0.8004 |  |
| Moderate SPUI to Severe SPUI | 0.143 | 0.1144 | 0.1716 |  |
| Severe SPUI to No to mild SPUI | 0.000 | 0.000 | 0.200 |  |
| Severe SPUI to Moderate SPUI | 0.500 | 0.400 | 0.600 |  |
| Severe SPUI to Severe SPUI | 0.500 | 0.400 | 0.600 |  |
| **No HME** | | | | |
| No to mild SPUI to No to mild SPUI | 0.262 | 0.2092 | 0.3138 | [7,14] |
| No to mild SPUI to Moderate SPUI | 0.738 | 0.591 | 0.886 |  |
| No to mild SPUI to Severe SPUI | 0.000 | 0.000 | 0.200 |  |
| Moderate SPUI to No to Mild SPUI | 0.172 | 0.1373 | 0.2059 |  |
| Moderate SPUI to Moderate SPUI | 0.670 | 0.536 | 0.804 |  |
| Moderate SPUI to Severe SPUI | 0.158 | 0.127 | 0.190 |  |
| Severe SPUI to No to mild SPUI | 0.000 | 0 | 0.2 |  |
| Severe SPUI to Moderate SPUI | 0.452 | 0.361 | 0.542 |  |
| Severe SPUI to Severe SPUI | 0.548 | 0.439 | 0.658 |  |

Abbreviations: HME, heat and moisture exchanger; SPUI, sputum impact

## Supplementary Table 5: Model input parameters: Cost of infection

| **Cycle** | **Age** | **Value** | **Source** |
| --- | --- | --- | --- |
| 0 | 67 | ¥451754 | [31] |
| 1 | 68 | ¥451754 |  |
| 2 | 69 | ¥451754 |  |
| 3 | 70 | ¥451754 |  |
| 4 | 71 | ¥451754 |  |
| 5 | 72 | ¥451754 |  |
| 6 | 73 | ¥451754 |  |
| 7 | 74 | ¥451754 |  |
| 8 | 75 | ¥451754 |  |
| 9 | 76 | ¥451754 |  |
| 10 | 77 | ¥451754 |  |

Abbreviations: ¥, Japanese Yen

## Supplementary Table 6: Model input parameters: Index cost

| **Data reference period** | **Consumer price index (12-month average growth rate- base 2012 - %)** | **2022** | **2021** | **2020** | **2019** | **2018** | **2017** | **2016** | **2015** | **2014** | **2013** |
| --- | --- | --- | --- | --- | --- | --- | --- | --- | --- | --- | --- |
| **Dec-22** | -0.94% | 0.9906 | 0.9906 | 0.986043 | 0.985353 | 0.973627 | 0.973627 | 0.965449 | 0.965449 | 0.966414 | 0.969024 |
| **Dec-21** | 0.00% |  | 1.000 | 0.9954 | 0.994703 | 0.982866 | 0.982866 | 0.97461 | 0.97461 | 0.975585 | 0.978219 |
| **Dec-20** | -0.46% |  |  | 0.9954 | 0.994703 | 0.982866 | 0.982866 | 0.97461 | 0.97461 | 0.975585 | 0.978219 |
| **Dec-19** | -0.07% |  |  |  | 0.9993 | 0.987408 | 0.987408 | 0.979114 | 0.979114 | 0.980093 | 0.982739 |
| **Dec-18** | -1.19% |  |  |  |  | 0.9881 | 0.9881 | 0.9798 | 0.9798 | 0.98078 | 0.983428 |
| **Dec-17** | 0.00% |  |  |  |  |  | 1.000 | 0.9916 | 0.9916 | 0.992592 | 0.995272 |
| **Dec-16** | -0.84% |  |  |  |  |  |  | 0.9916 | 0.9916 | 0.992592 | 0.995272 |
| **Dec-15** | 0.00% |  |  |  |  |  |  |  | 1.000 | 1.001 | 1.003703 |
| **Dec-14** | 0.10% |  |  |  |  |  |  |  |  | 1.001 | 1.003703 |
| **Dec-13** | 0.27% |  |  |  |  |  |  |  |  |  | 1.0027 |
| **Dec-12** | 2.77% |  |  |  |  |  |  |  |  |  |  |
| **Dec-11** | 3.65% |  |  |  |  |  |  |  |  |  |  |
| **Dec-10** | 1.40% |  |  |  |  |  |  |  |  |  |  |
| **Dec-09** | -0.83% |  |  |  |  |  |  |  |  |  |  |
| **Dec-08** | 2.59% |  |  |  |  |  |  |  |  |  |  |
| **Dec-07** | 2.45% |  |  |  |  |  |  |  |  |  |  |

## Supplementary Table 7: Model primary outcomes of cost-effectiveness of second-generation HMEs vs no HME

|  | Second-generation HMEs | No HME |
| --- | --- | --- |
| QALYs | 4.3328 | 4.3079 |
| Total Costs | ¥22038,701 | ¥21034,644 |
| Incremental QALYs | 0.02 | |
| Incremental Costs | ¥1004057 | |
| ICER | ¥40319287 | |

Abbreviations: HME, heat and moisture exchanger; ICER, incremental cost-effectiveness ratio; QALY, quality-adjusted life year; ¥, Japanese Yen

## Supplementary Table 8: Scenario analysis for second-generation HMEs vs no HME: Using JPY 170,000 as the cost for no HME

|  | Second-generation HMEs | No HME |
| --- | --- | --- |
| QALYs | 4.3328 | 4.3079 |
| Total Costs | ¥22038701 | ¥21541961 |
| Incremental QALYs | 0.02 | |
| Incremental Costs | ¥496740 | |
| ICER | ¥19947286 | |

Abbreviations: HME, heat and moisture exchanger; ICER, incremental cost-effectiveness ratio; QALY, quality-adjusted life year; ¥, Japanese Yen

## Supplementary Figure 1: PSA cost-effectiveness scatter plot for second-generation HMEs vs no HMEs


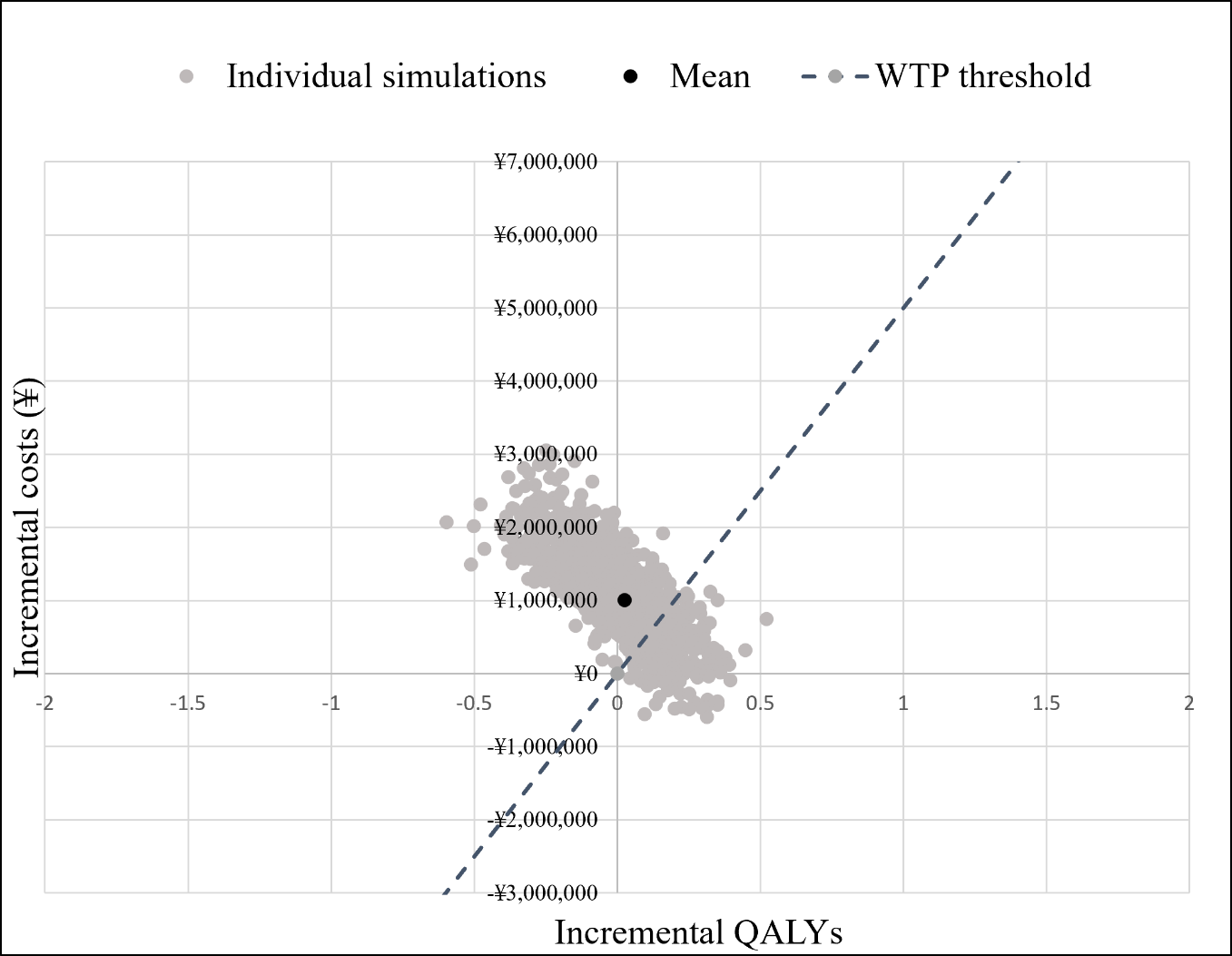


Abbreviations: HME, heat and moisture exchanger; PSA, Probabilistic Sensitivity Analysis; QALY, quality-adjusted life year; WTP, willingness to pay

## Supplementary Figure 2: Tornado plots displaying most influential parameters from one-way sensitivity analyses for second-generation HMEs versus no HME

Abbreviations: HME, heat and moisture exchanger; SPUI, sputum impact; ¥, Japanese Yen

## Supplementary Figure 3: Scenario analysis: Tornado plots displaying most influential parameters from one-way sensitivity analyses for second-generation HMEs versus no HME

Abbreviations: HME, heat and moisture exchanger; SPUI, sputum impact; ¥, Japanese Yen

## Supplementary data 1

### Primary and Secondary outcomes: Second-generation HMEs versus no HME

As compared to no HME, second-generation HMEs improved the QALY (4.33 vs 4.31) and increased the total healthcare spending per patient (JPY 22,038,701 vs JPY 21,034,644) over 10 years, resulting in an incremental QALY and incremental cost of 0.02 and, JPY 1,004,057 respectively (**Supplementary** **Table 7**). The resulting ICER (JPY 40,319,287 per QALY gained) was more than the WTP threshold value (JPY 5,000,000 per QALY gained), indicating that second-generation HMEs were not cost-effective than the no HME (**Supplementary** **Table 7**). Second-generation HMEs resulted in a fewer pulmonary infections (0.39 vs 0.55) and mucus plus event (0.47 vs 2.14), but led to an increased skin irritation (2.78 vs 0.00), compared to no HME (**Main manuscript Table 5)**.

### One-way sensitivity analysis: Second-generation HMEs versus no HME

Based on the OWSA, it was indicated that the transition probabilities between health states were the main drivers for the cost difference between second-generation HMEs and no HME (**Supplementary** **Figure 2)**.

### Scenario analysis for second-generation HMEs versus no HME: Using JPY 170,000 as the cost for no HME

Second-generation HMEs improved the QALY (4.33 vs 4.31) and increased the total healthcare spending per patient (JPY 22,038,701 vs JPY 21,541,961) as compared to no HME over 10 years, resulting in an incremental QALY and incremental cost of 0.02 and, JPY 496,740 respectively (**Supplementary** **Table 8**). The resulting ICER (JPY 19,947,286 per QALY gained) was more than the WTP threshold value (JPY 5,000,000 per QALY gained), indicating that second-generation HMEs were not cost-effective as compared to the no HME (**Supplementary** **Table 8**).

Based on the OWSA, it was indicated that the transition probabilities between health states were the main drivers for the cost difference between second-generation HMEs and no HME (**Supplementary** **Figure 3)**.
